# Novel Orientation-Sensitive Spin Probes for Graphene Oxide Membranes Study

**DOI:** 10.3390/membranes12121241

**Published:** 2022-12-08

**Authors:** Natalia A. Chumakova, Tamas Kalai, Anastasiya T. Rebrikova, Cecília Sár, Alexander I. Kokorin

**Affiliations:** 1 N.N. Semenov Federal Research Center for Chemical Physics, Russian Academy of Science, Kosygin St. 4, 119991 Moscow, Russia; 2 Department of Chemistry, Lomonosov Moscow State University, Leninskiye Gory, 1/3, 119991 Moscow, Russia; 3 Institute of Organic and Medicinal Chemistry, Faculty of Pharmacy, University of Pécs, Szigeti St. 12, H-7624 Pécs, Hungary; 4 Department of Chemistry, Plekhanov Russian University of Economics, Stremyanny per., 36, 115093 Moscow, Russia

**Keywords:** graphene oxide membrane, spin probe technique, orientational ordering

## Abstract

Spin probe EPR spectroscopy is currently the only method to quantitatively report on the orientational ordering of graphene oxide membranes. This technique is based on the analysis of EPR spectra of a membrane containing stable radicals sorbed on oxidized graphene planes. The efficiency of the method depends on the spin probe structure; therefore, it is important to find stable paramagnetic substances that are most sensitive to the alignment of graphene oxide membranes. In the present work, three novel stable nitroxide radicals containing aromatic fragments with two nitrogen atoms were tested as spin probes to study graphene oxide membranes. The spin-Hamiltonian parameters of the radicals in graphite oxide powder and orientational order parameters of the probes inside graphene oxide membrane were determined. The sensitivity of one of these radicals to membrane orientational ordering was found to be higher than for any of spin probes used previously. A likely reason for this higher sensitivity is the presence of heteroatoms which can facilitate interaction between paramagnetic molecules and oxygen-containing groups on the inner surface of the membrane. The new high-sensitivity spin probe may significantly increase the potential of EPR spectroscopy for studying the internal structure of graphene oxide membranes.

## 1. Introduction

Graphene oxide membranes (GOMs) are of great interest due to their selective permeability for different substances [1,2,3]. Presently, GOMs are considered as promising materials for water desalination and purification [4]. However, the physical reasons for the selective permeability of the membranes still need to be clarified. The authors of [1] suggested that the unique properties of GOMs may be due to their inner structure, specifically by the formation of a net of non-oxidized capillaries inside the membrane. This hypothesis seems probable since GOMs have a complex organization of oxidized graphene planes.

Cross-section SEM images showed that GOMs consist of lamellae oriented approximately along the membrane surface [5,6]; each lamella contains 15–30 oxidized graphene planes [7]. Until recently, the orientational alignment of GOMs was only studied visually based on cross-section SEM and TEM images. To our knowledge, there is only one paper focused on quantitative determination of the orientational ordering of the planes on the surface of the graphene oxide membranes using the polarized light microscopic images [8]. In our previous work [9,10] we proposed using the spin probe technique to characterize the orientation of the oxidized graphene planes inside the membranes. In this technique, stable paramagnetic molecules with high anisotropy of spin-Hamiltonian parameters are introduced into the inter-plane space of a graphene oxide membrane together with a polar liquid (water, acetonitrile, methanol, etc.). Then, the fluid is removed by drying the membrane in air and/or evacuation. The paramagnetic molecules—spin probes—are sorbed onto the oxidized graphene planes and remain in the membrane. A series of EPR spectra of the GOM containing spin probes are recorded at different membrane orientations in a magnetic field of an EPR spectrometer. Since paramagnetic molecules are sorbed on orientationally-ordered oxidized graphene planes, the spectra recorded at different orientations of the membrane in the magnetic field differ in shape. This difference—the angular dependence of EPR spectra—reflects the orientational ordering of the probes in the membrane, which, in turn, reflects the ordering of the planes. The orientation distribution function (ODF) of the radicals in the membrane is determined by simultaneous computer simulation of the spectral series [11].

ODF is the convolution of the ODF_micro_ and ODF_macro_, where ODF_micro_ reflects the orientation of the radicals relative to oxidized graphene planes, and ODF_macro_ means the orientation of the planes in the membrane. Obviously, the more ordered the radicals on the surface of the planes, the more accurately the ordering of the planes in the membrane is reported by ODF. Therefore, it is important to find stable paramagnetic substances that are most sensitive to the alignment of the membranes.

In [10], we tested different stable radicals (several nitroxides with different structures and [Cu(NH_3_)_4_]^2+^complex) as spin probes to investigate the alignment of the GOMs. It was shown that the orientational order parameters of the nitroxides and [Cu(NH_3_)_4_]^2+^ complex are comparable to each other, while rigid nitroxide radicals with aromatic fragments were found to be more sensitive. The next step of our study was to improve the sensitivity of the probes by varying the chemical structure of the aromatic fragments.

In the present work, we tested three novel stable nitroxide radicals, containing aromatic fragments with nitrogen atoms, as spin probes for studying the alignment of GOMs. We assumed that presence of heteroatoms in the aromatic rings can facilitate the interaction between paramagnetic molecules and oxygen-containing groups on the inner surface of the membrane, and, as a result, increase the orientation of the radicals in GOMs.

## 2. Materials and Methods

### 2.1. Materials

**Figure 1:**
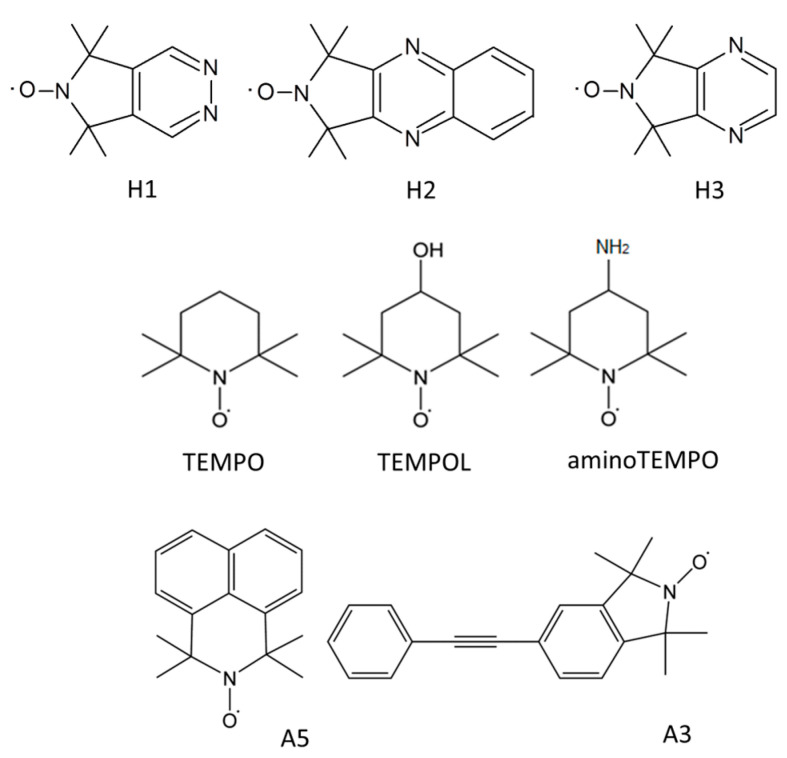
Structures of the novel spin probes and the probes used for studying GOMs earlier [10].

The structures of the novel nitroxide radicals are shown in Figure 1. The radicals were synthesized as described previously [12,13],

H1: 5,5,7,7-tetramethyl-5H-pyrrolo[3,4-d]pyridazin-6-yloxyl radical [12],

H2: 1,1,3,3-tetramethyl-1H-pyrrolo[3,4-b]quinoxalin-2-yloxyl radical [13],

H3: 5,5,7,7-tetramethyl-5H-pyrrolo[3,4-b]pyrazin-6-yloxyl radical [13].

Graphite oxide powder was purchased from ACS (CAS No.: 7782-42-5). The material was synthesized according the Hummers method [14]. GOM was prepared at Upsala University (Sweden). It should be noted that we used the same membrane as in [10] to study the orientational ordering of various spin probes in several GOMs. Therefore, we can compare the sensitivity of the novel probing molecules with the sensitivity of previously tested radicals.

Ultrapure toluene from Khimreactiv and HPLC acetonitrile from RCI Labscan were used as solvents.

### 2.2. Samples Preparation

The nitroxide spin probes were introduced into graphite oxide and graphene oxide membrane from acetonitrile. The radicals were dissolved in the liquid to form solutions with a concentration (0.01–0.05) mol/L. Five to six mg of powder or membrane fragments with a size of ~ 4 mm × 10 mm were kept in excess solutions for 6–7 days. Then, the samples were removed from the liquid and dried in air over 1–2 days. The prepared samples contained (1–5)·10^17^ spins per 1 mg of graphite oxide. Such concentrations permit avoiding spin-exchange and dipole-dipole interactions of probing molecules. Dry samples were kept in a desiccator with P_2_O_5_.

Solutions of the spin probes in toluene were degassed by five-fold repetition of the procedure “freezing—evacuated at 10^−5^ Torr—unfreezing”.

### 2.3. EPR Measurements

X-band EPR spectra were recorded using Bruker EMX-500 spectrometer with a high-sensitive resonator ER 4119 HS. Microwave power was chosen to avoid saturation of the EPR signals. The corresponding value was 1 mW at 298 K (room temperature) and (0.3–0.5) mW at 90–120 K.

The spectra of the acetonitrile solutions of radicals were recorded at 298 K in glass tubes with an inner diameter (*d*) of 1 mm. Spectra of the toluene solutions of radicals were recorded at 298 K, 120 K and 90 K in quartz ampules with *d* = 4.5 mm. Spectra of the “GO powder—spin probe” samples were recorded at 298 K, 120 K and 90 K in quartz ampoules with *d* = 2 mm. The temperature was controlled using the Bruker temperature control unit.

Spectra of the samples “GOM—spin probe” were recorded at 120 K using the home-made holder attached to the automatic Bruker goniometer. The holder makes it possible to turn the sample in the resonator and record spectra at different angles between the membrane surface normal and the magnetic field; the turning accuracy was 0.5°. A detailed description of the holder is given in [10]. For each sample, 13–15 spectra were recorded.

A simulation of the EPR spectra recorded at 90 K and 120 K was performed within the approximations of the “rigid limit” (no rotational mobility of radicals) and a weak external field excluding forbidden transitions. The orientation of principal axes of the g-tensor and the hyperfine interaction (HFI) tensor of the unpaired electron with the ^14^N were taken as collinear. The Voigt function described the shape of the individual resonance line. The contributions of the Gauss and Lorentz functions were described by tensors of the second rank with the principal axes coinciding with the magnetic axes. The software used is described in detail in [15,16,17]; it is available at http://www.chem.msu.ru/rus/lab/chemkin/ODF3 (accessed on 7 December 2022).

## 3. Results and Discussion

### 3.1. Spin-Hamiltonian Parameters of the Spin Probes

**Figure 2:**
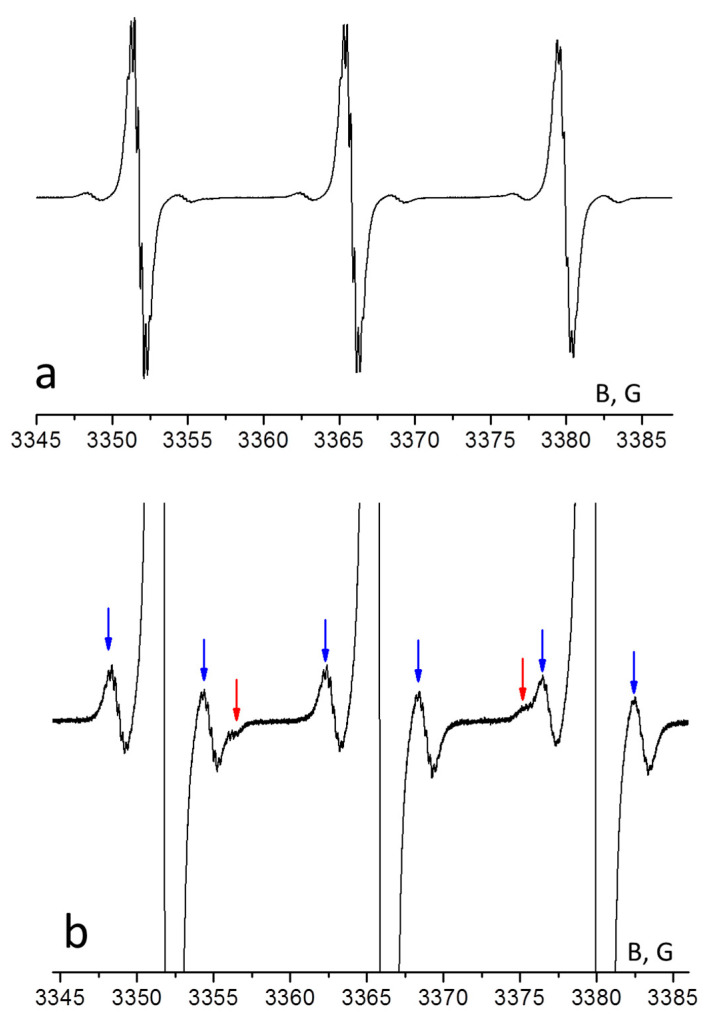
EPR spectrum of H1 in toluene at 298 K: (**a**)—full spectrum, (**b**)—satellite lines. The hyperfine lines of ^13^C and ^15^N are marked by blue and red arrows, correspondingly.

Figure 2 shows the EPR spectrum of radical H1 in toluene at room temperature. The spectra of H2 and H3 are similar to the spectrum of H1. The spectrum of H1 (Figure 2a) consists of three main lines caused by the hyperfine interaction of the unpaired electron with nucleus ^14^N of the paramagnetic nitroxide fragment. The satellite lines are well distinguished in Figure 2b. These lines reflect the splitting on nuclei ^13^C (the natural content is ca. 1.1%) and ^15^N (its natural content is ca. 0.37%) It should be noted that the spectrum of H1, as well as the spectra of H2 and H3, does not show any signal splitting on nuclei ^14^N located in aromatic fragments. Therefore, the spin density on aromatic nitrogen atoms is negligible. The isotropic magnetic parameters of H1, H2, and H3 in toluene are listed in Table 1.

For a more detailed investigation of the spin density distribution in the new spin probes, we performed quantum chemistry calculations of the optimized geometries and HFI constants using the DFT method in the ORCA program package [18]. Geometry optimization and HFI constant calculations were performed using (B3LYP, 3–61 g(d,p)) and (B3LYP, N07D) models, correspondingly. It is known that basis N07D has high forecasting power for hyperfine couplings in many paramagnetic substances, including nitroxide radicals [19]. It was found that the optimized geometry of all radicals are planar, and HFI on aromatic ^14^N does not exceed 0.1 G; such splitting cannot be observed in our experimental spectra. Therefore, EPR spectra of novel spin probes can be analyzed as the spectra of common nitroxide radicals.

**Figure 3:**
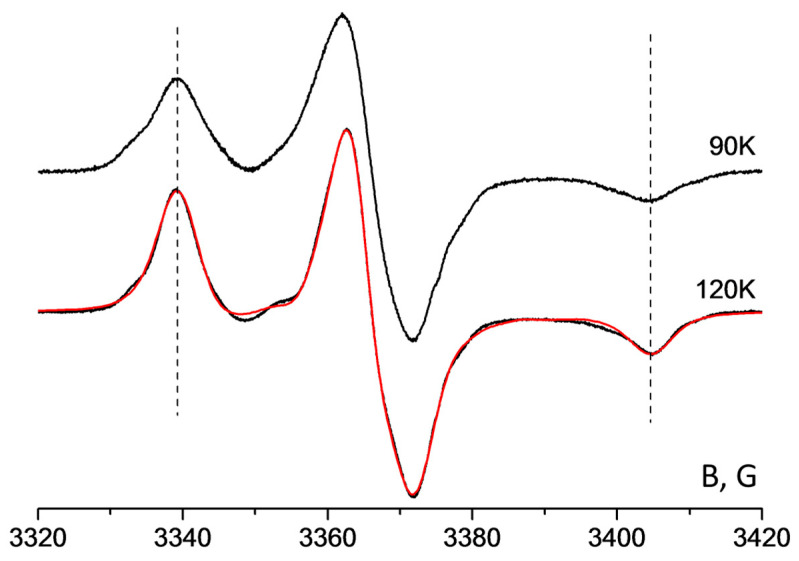
EPR spectra of H1 in frozen toluene. Black lines—experimental spectra, red line—simulation result.

The EPR spectra of H1 in frozen toluene are shown in Figure 3. The spectra of H2 and H3 in comparable samples are quite similar. It is seen that the positions of the low-field and high-field components of the spectra recorded at 90 K and 120 K coincide. This means that the rotational mobility of paramagnetic molecules at 120 K is insufficient for averaging the anisotropy of spin-Hamiltonian parameters. To determine the magnetic parameters, we have simulated the spectrum at 120 K which demonstrates narrower lines. The values of g- and HFI-tensors (A) of H1, H2, and H3 in toluene are given in Table 1.

**Figure 4:**
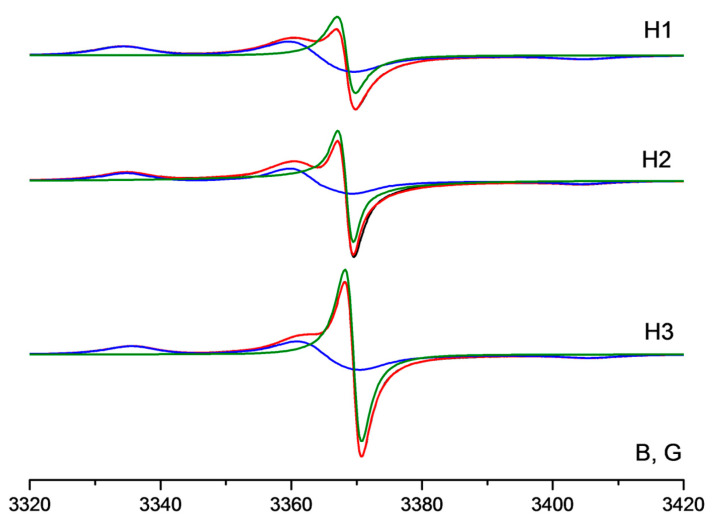
EPR spectra of H1, H2, and H3 in powder graphite oxide samples at 120 K. Black lines—experimental spectra, red lines—simulation results, blue lines—signals of nitroxide radicals, green lines—native signals of graphite oxide.

Figure 4 shows the EPR spectra of the novel nitroxide radicals in powder graphite oxide samples at 120 K. The intensive unstructured singlet line located in the central part of all EPR spectra belongs to a native signal of graphite oxide. The experimental EPR spectra were simulated as a sum of signals of the nitroxide radical and graphite oxide. Magnetic parameters of the spin probes sorbed on the inner surface of the material are also presented in Table 1.

It is well known that the A_zz_ parameter is sensitive to the polarity of the local environment of the nitroxide >N–O group [20]. It can be seen from of the data of Table 1 that the A_zz_ values for radicals H1, H2, and H3 sorbed on the oxidized graphene planes are much higher than the A_zz_ values for the radicals localized in nonpolar toluene. Thus, it can be concluded that the radicals inside graphite oxide are located mainly in the polar areas. Earlier it was experimentally shown that the distribution of oxygen-containing groups in the material is not random. Graphite oxide includes oxidized and non-oxidized (graphene) areas [21]. Evidently, the applied spin probes are localized primarily in the areas with the enlarged concentration of oxygen-containing groups.

### 3.2. Orientational Alignment of the Probing Molecules in Graphene Oxide Membrane

**Figure 5:**
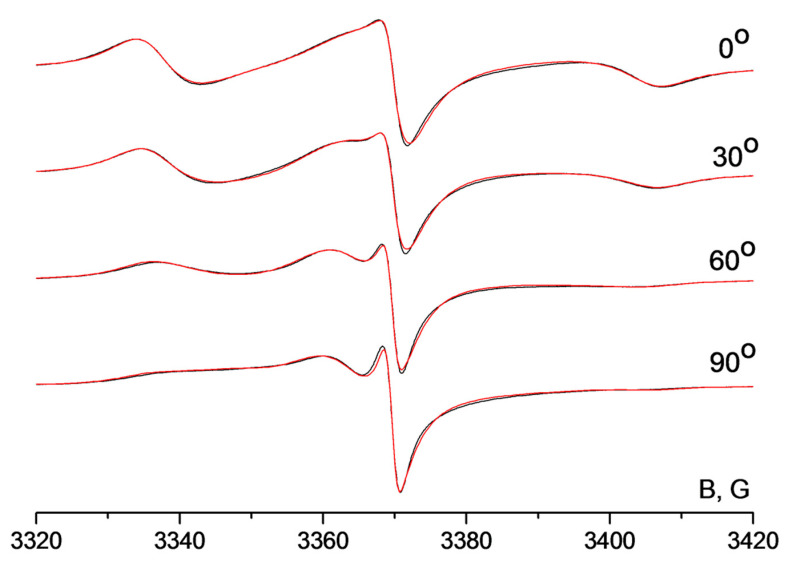
EPR spectra of the membrane including H1; the spectra were recorded at different angles between the membrane surface normal and magnetic field. Black lines—experimental spectra, red lines—result of simultaneous simulation of the spectral series.

The EPR spectra of the graphene oxide membrane spin-labeled by radical H1 are shown in Figure 5. Spectra were recorded at different angles between the membrane surface normal and the external magnetic field. One can see that the shape of the nitroxide signal changes significantly upon turning the membrane in the resonator of the spectrometer. Hence, spin probes in the membrane are ordered. For the determination of the orientation distribution function of the radicals in GOMs, we performed simultaneous simulation of the spectral series. The results of the simulation are shown in Figure 5. As an example, four spectra are shown in this Figure while we have recorded and simulated 18 spectra in total.

The orientational order parameters of the second and fourth ranks for radicals H1, H2, and H3 in GOM, which were determined from the simulations of the EPR spectra, are presented in Table 2. The order parameters are the averaged values of the spherical Legendre functions describing the orientation of the *Z*-axes of g-tensors of the radicals relatively to the membrane surface normal. A detailed description of the mathematical approach used to determine the orientational ordering of molecules in partially ordered systems is given in [11]. For comparison, Table 2 also contains the order parameters for other spin probes in the same membrane which were calculated previously [10]. The parameters listed in Table 2 describe the orientational ordering of different spin probes in the same membrane; therefore, higher values of the parameters mean higher sensitivity of the probing molecules to the alignment of the membrane.

The data obtained indicates that sensitivity of radicals H1 and H3 to the membrane alignment is practically the same as radicals A3 and A5 which also have aromatic moieties and much higher than sensitivity of the standard piperidine probes TEMPO, aminoTEMPO, and TEMPOL, as well as [Cu(NH_3_)_4_]^2+^ complex. At the same time, the sensitivity of the radical H2 significantly exceeds the sensitivity of all spin probes which were previously used for investigation of GOMs, including radical A5 which also contains two conjugated aromatic rings. We believe that the high sensitivity of H2 is related to the conjugated aromatic fragment with nitrogen atoms. Interaction of unhybridized orbitals of the nitrogen atoms with the oxygen-containing groups located on the surface of the oxidized graphene planes can significantly strengthen the interaction of the radicals with the inner surface of GOM and, as a result, improve the orientation of probing molecules in the membrane. Hence, radical H2 was found to have significant potential as a spin probe for studying the inner structure of GOMs.

## 4. Conclusions

Orientational ordering of the nitroxide spin probes in the graphene oxide membrane depends on the chemical structure of the probing molecules. The presence of a conjugated aromatic fragment containing two nitrogen atoms in the structure of a radical enhances its interaction with the oxygen-containing groups located on the surface of the oxidized graphene planes and, as a result, improves the orientation of the probing molecules in the membrane. The application of such spin probes increases the sensitivity of the spin probe technique to the study of alignment of graphene oxide membranes and makes it possible to investigate the behavior of the membranes under various treatments. The H2 spin probe was found to be more sensitive to the alignment of graphene oxide membranes compared with any of previously used spin probes.

## Figures and Tables

**Table 1:** Spin-Hamiltonian parameters of nitroxide radicals in toluene and on inner surface of graphite oxide.

	g_xx_	g_yy_	g_zz_	g_iso_	A_xx_	A_yy_	A_zz_	A_iso_
H1 toluene 298 K			2.0062				14.07	
H1 toluene 120 K	2.0095	2.0066	2.0024	2.0062	5.3	4.3	32.56	14.05
H1 GO 120 K	2.0092	2.0063	2.0024	2.0060	4.3	4.8	35.22	14.77
H2 toluene 298 K			2.0062				13.93	
H2 toluene 120 K	2.0095	2.0068	2.0024	2.0062	5.4	4.0	32.34	13.91
H2 GO 120 K	2.0092	2.0065	2.0024	2.0060	4.3	4.8	34.74	14.77
H3 toluene 298 K			2.0061				14.02	
H3 toluene 120 K	2.0094	2.0068	2.0023	2.0062	5.3	4.1	32.66	14.02
H3 GO 120 K	2.0092	2.0060	2.0024	2.0059	4.3	4.8	34.97	14.69

The uncertainties in determining of the g-values are 0.0002. The uncertainties in determining of the HFI-constants are 0.5 G for A_xx_, A_yy_, and 0.05 G for A_zz_, A_iso_.

**Table 2:** Orientational order parameters of various probing molecules in the same GOM.

Spin Probe	P_20_	P_40_
A3	0.41	0.23
A5	0.42	0.14
TEMPO	0.27	0.05
TEMPOL	0.31	0.07
4-aminoTEMPO	0.24	-
[Cu(NH_3_)_4_]^2+^	0.30	0.15
HR_2545	0.39	0.12
HR_4968	0.50	0.21
HR_4972	0.44	0.13

The uncertainties in determining the values of order parameters are 10–20% for P_20_, and 20–40% for P_40_. Order parameters of A3, A5, TEMPO, aminoTEMPO, TEMPOL, and [Cu(NH_3_)_4_]^2+^ complex were determined earlier in our work [10].

## Data Availability

Not applicable.
